# Characterization of implicit and explicit mind-reading in children with autism based on eye movements

**DOI:** 10.3389/fpsyt.2024.1449995

**Published:** 2024-10-30

**Authors:** Haidan Lu, Juanli Niu, Jiaxin Wang, Min Liu, Mingyu Xu

**Affiliations:** ^1^ Department of Rehabilitation of Sciences, Faculty of Education, East China Normal University, Shanghai, China; ^2^ Yangfan School, Shanghai, China; ^3^ School of Special Education, Suihua University, Changchun, China; ^4^ Department of Developmental and Behavioral Pediatric and Child Primary Care, Brain and Behavioral Research Unit of Shanghai Institute for Pediatric Research, Ministry of Education (MOE)-Shanghai Key Laboratory for Children's Environmental Health, Xinhua Hospital Affiliated to Shanghai Jiao Tong University School of Medicine, Shanghai, China

**Keywords:** children with autism, implicit mind-reading, explicit mind-reading, eye movements, false belief conditions

## Abstract

**Objective:**

This study aimed to investigate differences in mind-reading abilities between children with autism and typically developing children across various tasks.

**Methods:**

Sixteen children with autism (aged 5-8 years) were compared to 16 typically developing children matched in language ability. The unexpected location task and unexpected content task were used to assess implicit and explicit mind-reading abilities using an eye tracker and illustrated storybooks.

**Results:**

For implicit mind, using differential looking scores shows a no significant difference between the scores of children with autism and typically developing children in the implicit tasks (P=0.399). However, the pupil size show some significant difference between two groups. Second, for the explicit, a significant difference between the scores of children with autism and typically developing children in the explicit tasks (P=0.006). Additionally, only a significant correlation between implicit and explicit mind-reading abilities in children with autism in the unexpected location task was found.

**Conclusion:**

The mechanism of implicit mind-reading still not very clear. Pupil-Linked arousal response can be consider as a further tool. Further research on implicit and explicit mind-reading abilities is warranted.

## 1 Introduction

Theory of mind (ToM), also known as mind-reading, refers to the ability to perceive one’s own and others’ mental states (thoughts, intentions, emotions, beliefs, etc.) and to make consequential predictions as well as explanations of corresponding behaviors (1). It involves understanding others’ mental states and plays a vital role in social interactions and communication (2). Apperly and Butterfill (3) proposed a “two-systems theory” of mind, which argues that mind-reading involves an implicit system and an explicit system. Implicit theory of mind is a rapid, unconscious, automated way of processing others’ mental states that operates without direct words or instructions (4). Reseachers also think implicit mind-reading may also a promising account for autism and highly link with the contexts for implicit mentalizing (5). Conversely, the ability to consciously recognize one’s own or another person’s mental state and to causally explain and predict behavior based on this cognition is referred to as explicit theory of mind (6). Research on children’s explicit mind-reading has found that typically developing children can understand false beliefs around the age of 4, while infants and toddlers exhibit the capacity for implicit mind-reading by approximately 7-18 months of age (7).

The most common method for assessing children’s ToM is the false belief test, which evaluates a child’s ability to realize that others can hold different or false ideas or beliefs (8). Testing specifically encompasses paradigms such as unexpected location tasks and unexpected content tasks (9). An initial exploration of children’s understanding of false beliefs involved elicited-response tasks, where children were asked to verbalize answers regarding false beliefs (10). It has been shown that typically developing children can successfully complete explicit elicited-response tasks around age 4, while children with autism complete the task without considering the protagonist’s false beliefs, a finding confirmed across different false belief tests (11).

Non-verbal spontaneous-response tasks are increasingly used to assess implicit mind-reading. Unlike elicited-response tasks, these tasks do not directly ask whether the subject will engage in certain behaviors but rather record spontaneous behaviors such as gaze shifts, differences in gaze duration, and gaze preferences after observing performers’ behaviors (12). Senju et al. (13) examined the implicit ToM of autistic children aged 6-8 years using a spontaneous-response unexpected location task and found that, even when controlling for language, children with autism struggled to maintain predictive gaze at the correct location as typical children do, indicating impairment in implicit ToM. Hirshkowitz and Rutherford (14) use eye tracking procedure as an evidence of an appreciation of FB at 7 months.

However, only eye tracking seems not enough to explain how implicit mind happen and how it works with explicit mind. Recently, not only gaze also pupillometry holds potential as an unobtrusive way to measure the cognitive effort associated with a given task (15–17). Pupillometry may even be more reliable than behavioral measures in perception and other mental responses. Tortelli et al. (18) found that higher Autism-Spectrum Quotient(AQ)’s pupillary modulation was larger for human participants, with attention focused on the front surface. Per Bækgaard et al. (19) using an assembly task found that children who were least productive and asked for assistance more often had a significantly different pupil pattern than the rest. Prochazkova et al. (20) thought human pupil mimicry modulates trust decisions through the activation of the theory-of-mind network. However, how pupillometry can exactly explain different groups cognitive still needs more discussion as the pupil size will affected by the light in a fence and task complicacy. A meta-analysis of Autism spectrum disorder and pupillometry show that pupillometry reveals differences between people with and without ASD. The evidence on baseline pupil size and amplitude change is conflicting, the exact meaning of these differences remains unknown. So future studies should align research designs and investigate a possible effect of maturation (21).

Research suggests that social interaction and communication difficulties in children with autism may stem from impairments in mind-reading (22). And separately investigate often got controversial conclusions (23). Though some researchers have been consider about the relationship between implicit and explicit mind-reading, most design for adults (24, 25). And implicit mentalizing is more strongly associated with objectively measured correlates. These findings underscore the importance of an integrative approach considering both implicit and explicit mentalizing (26). Accordingly, this study aims to use more eye tracking characterizes and explore the relationship between implicit and explicit mind-reading abilities in children with autism using two false belief tests: the unexpected location task and the unexpected content task. This research aims to inform theoretical studies and clinical practice regarding the mind-reading abilities of children with autism.

## 2 Methods

### 2.1 Participants

Autistic children and typically developing children matched in language skills were randomly selected from a kindergarten in Jiaxing City. The inclusion criteria for autistic children were: (1) having an autism identification report issued by a hospital; (2) having a common language of Mandarin Chinese, being able to communicate simply with the tester, and having a language age of 4 years or older as assessed by the Peabody Picture Vocabulary Test Revised (PPVT-R); (3) no significant emotional or behavioral problems and the ability to sit peacefully for more than 10 minutes; (4) no other impairments such as hearing impairment or visual impairment. Eventually, 16 children with autism participated in the study: 11 boys (69%) and 5 girls (31%). The inclusion criteria for typical children were: (1) common language was Mandarin Chinese, and the language age matched that of the autistic child subjects as assessed by the PPVT-R; and (2) exclusion of other types of disorders such as hearing impairment and autism. Eventually, 16 typically developing children participated in the study: 8 boys (50%) and 8 girls (50%). There was no significant difference in PPVT-R scores between the two groups by independent samples t-test (t=1.988, P=0.056). Consent for participation was obtained from the parents and teachers of the subjects. The basic information of the subjects is shown in **Table 1**.

**Table 1:** Subject’s basic information.

Groups	Typical children (*n*=16)	Autistic children (*n*=16)
Physiological age	5-6	6-8
PPVT-R	92.88 ± 5.28	76.13 ± 6.56

### 2.2 Materials and equipment

**Figure 1:**
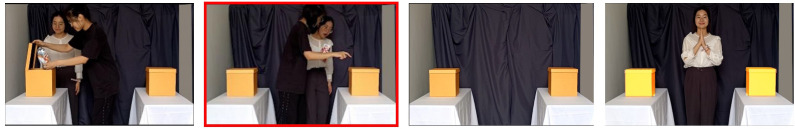
Unexpected location task - witnessed phase.

**Figure 2:**
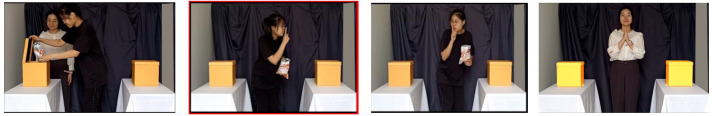
Unexpected location task - unwitnessed phase.

**Figure 3:**
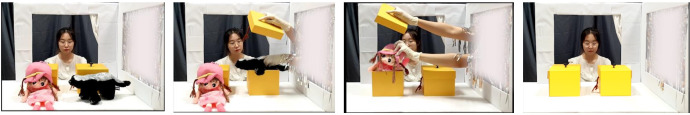
Unexpected content task - witnessed phase.

**Figure 4:**
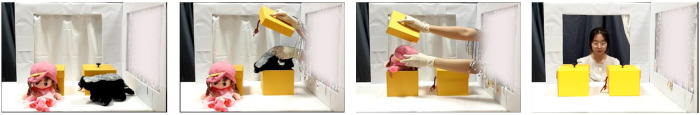
Unexpected content task - unwitnessed phase.

Video material was filmed with reference to Kulke’s modified unexpected location video task as well as Song’s unexpected content video task, and subjects’ implicit mind-reading abilities were assessed using an eye tracker. The experimental phase was divided into two conditions: correct and false beliefs. The experimental paradigm for the unexpected location task is the anticipatory gaze paradigm (25); differential looking scores (DLS) were calculated by recording the duration of subjects’ gaze on the target area. The DLS is calculated as the ratio of the subject’s gaze duration looking at the belief-consistent box minus the gaze duration looking at the belief-inconsistent box to the total gaze duration looking at the two boxes, which ranges between -1 and 1. A higher value indicates a better ability to understand false beliefs, as shown in **Figures 1**, **2**. The experimental paradigm for the unexpected content task is the violation of expectation paradigm (27); the average pupil size of the subjects was counted while watching the paused frame of the video, as shown in **Figures 3**, **4**. Participants’ gaze duration within 25 seconds in the two conditions of the correct belief that the actress had witnessed the situation and false belief that she had not witnessed the situation was measured, and also calculated the average pupil size within 25 seconds. Additionally, a standard false-belief task adapted to the unexpected location and unexpected content tasks examined subjects’ explicit mind-reading ability, referencing Baron-Cohen et al., where subjects’ responses to questions about false beliefs were scored on a scale of 0 to 1 (9).

The video was captured using a Huawei cell phone and edited in Adobe Premiere Pro 2022. Eye movement data were recorded by an eye tracker (Tobii Pro Fusion Eye Tracker Unit 250Hz), sampling gaze position at a frequency of 250 Hz using Tobii Pro Lab software (version 1.207). The stimulus video was presented on a Hewlett-Packard (HP) laptop (i5-11400H 16G 512GSSD RTX3050Ti) with a 16.5-inch (42cm, 1920 x 1080 pixels) screen. Subjects were approximately 60-70 cm from the monitor.

### 2.3 Procedures

The implicit unexpected location task was divided into a familiarization phase and two experimental phases. The familiarization phase was designed for subjects to build the belief that the actress intended to take the snack, and the experimental phase was divided into two conditions: correct beliefs when the actress witnessed the event, and false beliefs when the actress did not (the video was approximately 60-73 seconds long). The implicit unexpected content task was divided into a familiarization phase, a box orientation phase, and four experimental phases. The familiarization phase was designed for subjects to build the belief that the actress liked the doll and wanted to hold it, while the box orientation phase confirmed that a strand of the doll’s braid was attached to the lid of one of the boxes. The experimental phase was similarly divided into correct and false belief conditions (the video lasted approximately 62 seconds), with each condition featuring a normal box and a camouflaged box event. To ensure accurate baseline pupil size measurements, participants were instructed to fixate on a central point on a blank screen for 60 seconds under uniform lighting conditions. The average pupil diameter during this period was calculated for each participant and used as their baseline measure. During the experimental phase, the average pupil size recorded in the last 25 seconds of video viewing was determined. Pupil size recordings were aligned to the materials each type of perceptual phase (corresponding to zero time), from which the average pupil size in the last 6 seconds immediately following stopped. The change in pupil size was computed as the difference between this average and the baseline value. This differential measure was then used for subsequent analyses.

The explicit unexpected location task required subjects to predict where the storyteller would look for an item after being informed of its relocation, despite the storyteller’s ignorance of this change. The explicit unexpected content task involved presenting subjects with a container whose outward appearance suggested specific contents, then revealing the actual different contents, and subsequently asking subjects to predict others’ assumptions about the container’s contents. Sally-Anne test paradigm was used as unexpected location task and Smarties box test was used as unexpected content task. For Sally-Anne test mode, we use bunny and panda as the characters. And for the Smarties box, we use a candy box with a pen. All the materials are painted, and printed as a story book.

Both implicit and explicit test use “one-on-one” administration method. The tester selected a specified video and instructed, “Today, let’s watch a few small videos together, and keep quiet while watching them.” After the video, the subjects were shown picture cards and instructed, “Let’s watch two stories together, and after the teacher has finished, please come and answer a few questions from the teacher.” Upon completing the test, subjects received social reinforcement or stickers as a reward. The entire test took approximately 45-55 minutes.

### 2.4 Data analysis

Descriptive statistics, independent samples t-test, chi-square test and correlation analysis were performed using SPSS 23.0.

## 3 Results

### 3.1 Results of eye movement analysis during an implicit task in two groups of children

#### 3.1.1 Behavior in the implicit unexpected location task

The results of an independent samples nonparametric test with subject types and experimental conditions as independent variables, and DLS as the dependent variable, are shown in **Table 2**. The DLS was significantly higher in typically developing children than in children with autism in the implicit unexpected location task under the correct belief condition (P=0.020). However, there was no significant difference in DLS between the two groups under the false belief condition (P=0.705). The mean DLS for typically developing children was greater than 0 and greater than that of children with autism.

**Table 2:** A test of differences in implicit unexpected location task between two groups.

Experimental condition	Typical children	Autistic children	*Z*	*P*
	*M ± SD*	*M ± SD*		
Correct belief	0.44 ± 0.51	-0.22 ± 0.83	-2.332	0.020*
False belief	0.02 ± 0.65	-0.06 ± 0.70	-0.378	0.705

*P<0.05.

#### 3.1.2 Behavior in the implicit unexpected content task

An independent samples t-test was conducted with subject types and experimental conditions as independent variables, and the mean pupil size of subjects gazing at the paused video screen as the dependent variable. The results are shown in **Table 3**. There was a significant difference in the mean pupil size of typically developing children when they gazed at the normal box event and the camouflaged box event in the implicit unexpected content task under the correct belief condition (P=0.035). The pupil size was larger when they gazed at the camouflaged box event video. In the false belief condition, there was a significant difference in the mean pupil size of typically developing children when they gazed at the normal box event video and the camouflaged box event video (P=0.026), with the pupil size being larger for the normal box event video. However, there was no significant difference in the mean pupil size of children with autism when viewing the two event videos in either the correct or false belief conditions (P>0.05).

**Table 3:** A test of differences mean pupil size in implicit unexpected content task between two groups.

Experimental condition		Typical children	*P*	*t*	Autistic children	*P*	*t*
		*M ± SD*			*M ± SD*		
Correct belief	Normal box	3.96 ± 0.48	0.035*	-2.316	4.11 ± 0.64	0.598	0.539
	Camouflaged box	4.07 ± 0.51			4.07 ± 0.48		
False belief	Normal box	4.08 ± 0.47	0.026*	2.462	4.11 ± 0.48	0.272	-1.140
	Camouflaged box	4.00 ± 0.48			4.17 ± 0.55		

*P<0.05.

#### 3.1.3 Total scores for implicit tasks

To examine the overall differences in implicit mind-reading abilities between children with autism and typically developing children on different tasks, subjects were scored on a 0 to 1 scale based on their gaze discrepancy scores on the implicit unexpected location task and their mean pupil size on the implicit unexpected content task. The scores were summed to give the subject’s total score on the implicit tasks. A chi-square test was conducted with subject types and experimental conditions as independent variables and the total score of the implicit tasks as the dependent variable. The results are shown in **Table 4**, indicating no significant difference between the scores of children with autism and typically developing children in the implicit tasks (P=0.399).

**Table 4:** Results of the chi-square test for the total scores on the implicit task in two groups.

Experimental condition	Typical children			Autistic children			Precise significance
	Number of 0 points	Number of 1 points	Number of 2 points	Number of 0 points	Number of 1 points	Number of 2 points	
Implicit task	3	6	7	5	8	3	0.399

*P*>0.01.

### 3.2 Results of the scores on picture stories during an explicit task in two groups of children

#### 3.2.1 Behavior in the explicit unexpected location task

Using the type of subjects and experimental conditions as independent variables, the subjects’ responses to the question “When the bunny comes back to the room, where does it go to look for candy?” were scored on a 0 to 1 scale as the dependent variable for the chi-square test. The results are shown in **Table 5**. There was a highly significant difference between the scores of children with autism and typically developing children on the explicit unexpected location task (P=0.004). Thirteen typically developing children were correct, significantly more than the 4 correct children with autism.

**Table 5:** Results of the chi-square test for scores on the explicit task in both groups.

Condition	Tasks	Typical children			Autistic children			Precise significance
		Number of 0 points	Number of 1 points	Number of 2 points	Number of 0 points	Number of 1 points	Number of 2 points	
False belief	Unexpected location task	3	13		12	4		0.004**
	Unexpected content task	0	16		9	7		0.001**
	Total scores	0	3	13	7	7	2	0.006**

**P<0.01.

#### 3.2.2 Behavior in the explicit unexpected content task

Using the type of subjects and experimental conditions as independent variables, the subjects’ responses to the question “What does the little panda think is in the box?” were scored on a 0 to 1 scale as the dependent variable for the chi-square test. The results are shown in **Table 5**. There was a highly significant difference between the scores of children with autism and typically developing children on the explicit unexpected content task (P=0.001). Sixteen typically developing children were correct, significantly more than the 7 correct children with autism.

#### 3.2.3 Total scores for explicit tasks

To examine the overall differences between children with autism and typically developing children’s explicit mind-reading abilities on different tasks, the subjects’ scores on the explicit unexpected location task and the explicit unexpected content task were summed to produce their total explicit task scores. A chi-square test was conducted with subject types and experimental conditions as independent variables and the total number of explicit task scores as the dependent variable. The results are shown in **Table 5**. There was a significant difference between the scores of children with autism and typically developing children in the explicit tasks (P=0.006). In both tasks, more typically developing children were correct than children with autism.

### 3.3 The relationship between implicit and explicit mind-reading in children with autism

To investigate the relationship between implicit and explicit mind-reading in children with autism, Kendall’s correlation analyses were conducted on the subjects’ total scores on the implicit task and total scores on the explicit task. The results are shown in **Table 6**. There was a significant positive correlation between scores on the implicit unexpected location task and scores on the explicit unexpected location task for children with autism (r=0.560, P=0.024). There was not a significant positive correlation between scores on the implicit unexpected content task and scores on the explicit unexpected content task (r=0.492, P=0.057), but the p-value was close to 0.05. These results suggest that implicit mind-reading ability is positively correlated with explicit mind-reading ability in an unexpected location task for children with autism.

**Table 6:** Results of correlation analysis of scores on different tasks in children with autism.

	Implicit unexpected location task	Implicit unexpected content task
Explicit unexpected location task	0.560*	0.364
Explicit unexpected content task	0.270	0.492

*P<0.05.

## 4 Discussion

In this study, we found that children with autism exhibit varied performance in mind-reading ability across different task contents (unexpected location task and unexpected content task) and task levels (implicit and explicit), all of which significantly lag behind typically developing children.

### 4.1 Implicit tasks

First, in the implicit unexpected location task, children with autism had significantly lower gaze discrepancy scores than typically developing children in the correct belief condition. This suggests that children with autism had more difficulty gazing at the box after the snack had been shifted, whereas typically developing children were able to comprehend that when the actress returned to the scene, she would seek the snack based on its known new location. In contrast, in the false belief condition, there was no significant difference in gaze discrepancy scores between typically developing children and children with autism. This may be because, in this experimental condition, actress 2 first transferred the snack to the other box and then took it away from the scene without being witnessed by actress 1. There was no snack in either box, and this higher memory load posed challenges that may impair children’s ability to follow actors (13). Additionally, subjects may have been influenced by the video of the correct belief condition, as their learning effects interfered with spontaneous responses in the false belief condition. Future research could further improve experimental design by randomizing the experimental arrangement.

In the implicit unexpected content task, we instead focus on the simpler concept of averaged means of the pupil size. Some research noticed that the averaged means can reflects not only phasic responses but rather a combination of the current tonic level (arousal) and any phasic activations that take place. Hyönä et al. (28) considered it can be a concept as an index into a combined level of cognitive effort, as global processing load or the pupillometric estimate of mental load. Similar ideas have also been proposed elsewhere (29, 30). Typically developing children showed a significant pupil enlargement response when they saw outcomes that violated their expectations, as indicated by the mean pupil size. Under the correct belief condition, the actress should have opened the normal box to get her favorite doll toy, but unexpectedly, she opened the camouflaged box with the hair attached to it. In the false belief condition, the actress didn’t see the doll toy being placed in the normal box, but still opened the normal box unexpectedly. Erstenyuk administered a free-viewing joint attention task designed to elicit gaze following in 39 autistic children aged 3-9 years. Findings showed a negative correlation between pupil dilation and parent-reported subclinical symptoms of autism. This means that children with higher levels of autism-related symptoms exhibited less pupil dilation on a joint attention task when gaze cues did not correspond to the target location, which was positively correlated with Social Responsiveness Scale (SRS) summary scores. These results suggest that children with fewer autism-related symptoms allocate more cognitive resources (31). This data analysis of mean pupil change could be added to future implicit studies to better identify the mind-reading abilities of impaired groups.

However, several researches has been noticed that more complex visual stimuli, which can contain social emotional information, compare familiar and unfamiliar information (32) require local or global processing, and may involve active processing strategies. It’s a limitation for the pupil size lack a check of the differences in baseline pupil dilation, which might affect the main results. In the future, baseline and more critical frame need to be consider.

In summary, children with autism scored significantly lower than typically developing children under the correct belief condition of the implicit unexpected location task and under both correct and false belief conditions of the implicit unexpected content task, regardless of the box the actress was expected to check for the snack or the doll toy. This reflects that children with autism do have difficulties in social interaction and communication and have difficulty in understanding the psychological state of others.

### 4.2 Explicit tasks

Huang (33) measured autistic children’s performances in six tasks, including appearance-reality, conflict-true beliefs, and unexpected location. The results showed that autistic children’s abilities in different tasks and the overall tasks significantly lagged behind typically developing children. The present study also found that children with autism performed significantly lower than typically developing children on explicit tasks such as predicting where the bunny would look for candy and what items the panda thought were in the box. These findings are consistent with previous research and further suggest that the development of mind-reading abilities may be delayed or abnormal in the autism population.

### 4.3 The relationship between implicit and explicit mind-reading abilities

Finally, in the correlation comparison between implicit and explicit tasks, there was a significant positive correlation between the scores of children with autism on the implicit unexpected location task and the scores on the explicit unexpected location task. This suggests that children with autism show a correlation between their implicit mind-reading ability and their explicit mind-reading ability on the unexpected location task. This is similar to previous findings that implicit theories of mind are significantly correlated with explicit theories of mind and that both systems are influenced by commonly required cognitive resources (6). Low (34) tested the same subjects’ performance on an explicit unexpected location task, an explicit unexpected content task, and a deception-appearance task, finding that performance on implicit tasks predicted performance on explicit tasks. These studies consistently show a correlation between implicit and explicit mind-reading abilities in children with autism, providing a theoretical basis for subsequent interventions in mind-reading abilities. However, researchers still need to be cautious self-report and behavioral measures of the same construct were weakly correlated (35). More recently, people think what others’ know, hear, see, are more likely candidates for implicit ToM representations.

## 5 Conclusion

Based on the teaching and developmental needs of children with autism, this study centered on the relationship between implicit and explicit mind-reading in children with autism across different tasks from a new perspective, comparing them to typically developing children with matched language age. It was found that five out of six experimental tasks showed that children with autism lagged behind typically developing children with matched language skills in their mind-reading abilities. Regarding implicit mind-reading ability, three of the four experiments showed significant or highly significant differences between the performance of children with autism and typically developing children. The exception was the false-belief condition of the unexpected location task, where no significant difference was observed. Regarding explicit mind-reading ability, the scores of children with autism differed significantly from typically developing children in both the unexpected location and unexpected content tasks. There was also a significant positive correlation between scores on the implicit unexpected location task and scores on the explicit unexpected location task in children with autism.

This study also has some shortcomings and limitations. First, regarding the number of subjects, due to the specificity of the autism disorder group and the complexity of the task, the sample size is not large enough, which may influence the final results. Future studies can further increase the sample size. Secondly, for the pupil size, should consider more light and baseline in the future. And at the task level, this study focuses on the different performance of children with autism and typically developing children on the unexpected location and unexpected content tasks. Subsequent studies may further explore the effects of task difficulty, individual language level, and age on children’s mind-reading ability. Lastly, regarding impairment categories, this study was limited to comparing the mind-reading abilities of two groups: typically developing children and children with autism. Other groups, such as those with hearing impairments and intellectual disabilities, could be added for comparison in future experiments.

## Data Availability

The original contributions presented in the study are included in the article/supplementary material. Further inquiries can be directed to the corresponding author.

## References

[1] Premack D Woodruff G. Does the chimpanzee have a theory of mind? Behav Brain Sci. (1978) 1:515–26. doi: 10.1017/S0140525X00076512

[2] Waytz A Gray K Epley N Wegner DM. Causes and consequences of mind perception. Trends Cogn Sci. (2010) 14:383–8. doi: 10.1016/j.tics.2010.05.006 20579932

[3] Apperly IA Butterfill SA. Do humans have two systems to track beliefs and belief-like states? psychol Rev. (2009) 116:953–70. doi: 10.1037/a0016923 19839692

[4] Liu S. A study of the developmind characteristics of deaf students’ implicit theory of mind. Master’s thesis, Hunan: Hunan Normal University (2021).

[5] Wu R Leow K Yu N Rafter C Rosenbaum K Hamilton AFdeC. Evaluative contexts facilitate implicit mentalizing: relation to the broader autism phenotype and mental health. Sci Rep. (2024) 14:4697. doi: 10.1038/s41598-024-55075-9 38409351 PMC10897468

[6] Chen J. The relationship between implicit and explicit theories of mind in adults: the influence of cognitive resources. Master’s thesis, Changchun: Northeast Normal University (2019).

[7] Onishi KH Baillargeon R. Do 15-month-old infants understand false beliefs? Science. (2005) 308:255–8. doi: 10.1126/science.1107621 PMC3357322 15821091

[8] Peters KA Beer J Pisoni D Remmell E. Theory of mind acquisition in children who are deaf: The importance of early identification and communication access. J Early Hearing Detection Intervention. (2021) 6:101–13. doi: 10.26077/F2F7-49A9

[9] Baron-Cohen S Leslie AM Frith U. Does the autistic child have a “theory of mind”? Cognition. (1985) 21:37–46. doi: 10.1016/0010-0277(85)90022-8 2934210

[10] Baillargeon R Scott RM He Z. False-belief understanding in infants. Trends Cogn Sci. (2010) 14:110–8. doi: 10.1016/j.tics.2009.12.006 PMC2930901 20106714

[11] Perner J Leekam SR Wimmer H. Three-year-olds’ difficulty with false belief: The case for a conceptual deficit. Br J developmind Psychol. (1987) 5:125–37. doi: 10.1111/j.2044-835X.1987.tb01048.x

[12] Kulke L Hinrichs MAB. Implicit theory of mind under realistic social circumstances measured with mobile eye-tracking. Sci Rep. (2021) 11:1215. doi: 10.1038/s41598-020-80614-5 33441890 PMC7806733

[13] Senju A Southgate V Miura Y Matsui T Hasegawa T Tojo Y. Absence of spontaneous action anticipation by false belief attribution in children with autism spectrum disorder. Dev Psychopathol. (2010) 22:353–60. doi: 10.1017/S0954579410000106 20423546

[14] Hirshkowitz A Rutherford MD. Longer looking to agent with false belief at 7 but not 6 months of age. Infant Child Dev. (2021) 30:e2263. doi: 10.1002/icd.2263 35864890 PMC9286622

[15] Turi M Burr DC Binda P. Pupillometry reveals perceptual differences that are tightly linked to autistic traits in typical adults. eLife. (2018) 7:e32399. doi: 10.7554/eLife.32399 29506652 PMC5839694

[16] Pomè A Burr DC Binda P. Spontaneous pupillary oscillations increase during mindfulness meditation. Curr Biol. (2020) 30:R1030. doi: 10.1016/j.cub.2020.07.064 32961153

[17] Tortelli C Turi M Burr DC Binda P. Pupillary responses obey emmert's law and co-vary with autistic traits. J Autism Dev Disord. (2021) 51:2908–19. doi: 10.1007/s10803-020-04718-7 PMC8254720 33089444

[18] Tortelli C Turi M Burr DC Binda P. Objective pupillometry shows that perceptual styles covary with autistic-like personality traits. eLife Sci. (2021) 10:e67185. doi: 10.7554/eLife.67185 PMC8016475 33749589

[19] Bækgaard P Jalaliniya S Hansen JP. Pupillary measurement during an assembly task. Appl Ergonomics. (2019) 75:99–107. doi: 10.1016/j.apergo.2018.09.004 30509543

[20] Prochazkova E Prochazkova L Giffin MR Scholte HS De Dreu CKW Kret ME. Pupil mimicry promotes trust through the theory-of-mind network. Proc Natl Acad Sci USA. (2018) 15:201803916. doi: 10.1073/pnas.1815545115 PMC6077696 30012623

[21] De Vries L Fouquaet I Boets B Naulaers G Steyaert J. Autism spectrum disorder and pupillometry: a systematic review and meta-analysis. Neurosci Biobehav Rev. (2021) 120:479–508. doi: 10.1016/j.neubiorev.2020.09.032 33172600

[22] Senju A. Spontaneous theory of mind and its absence in autism spectrum disorders. Neuroscientist. (2012) 18:108–13. doi: 10.1177/1073858410397208 PMC3796729 21609942

[23] Ben-Shalom D Mostofsky SH Hazlett RL Goldberg MC Landa RJ Faran Y. Normal physiological emotions but differences in expression of conscious feelings in children with high-functioning autism. J.Autism Dev Disord. (2006) 36:395ord.m. doi: 10.1007/s10803-006-0077-2 16565884

[24] Nicholson T Williams DM Grainger C Lind SE Carruthers P. Relationships between implicit and explicit uncertainty monitoring and mindreading: Evidence from autism spectrum disorder. Conscious Cogn. (2019) 70:11–24. doi: 10.1016/j.concog.2019.01.013 30776592

[25] Kulke L Wübker M Rakoczy H. Is implicit Theory of Mind real but hard to detect? Testing adults with different stimulus materials. R Soc Open Sci. (2019) 6:190068. doi: 10.1098/rsos.190068 31417713 PMC6689622

[26] Kivity Y Levy KN Johnson BN Rosenstein LK LeBreton JM. Mentalizing in and out of awareness: A meta-analytic review of implicit and explicit mentalizing. Clin Psychol Rev. (2024) 108:102395. doi: 10.1016/j.cpr.2024.102395 38320421

[27] Song HJ Baillargeon R. Infants’ reasoning about others’ false perceptions. Developmind Psychol. (2008) 44:1789. doi: 10.1037/a0013774 PMC3372913 18999340

[28] Hyönä J Tommola J Alaja A-M. Pupil dilation as a measure of processing load in simultaneous interpretation and other language tasks. Q J Exp Psychol Section A. (1995) 48:598. doi: 10.1080/14640749508401407 7568993

[29] Iqbal ST Zheng XS Bailey BP. (2004). Task-evoked pupillary response to mental workload in human-computer interaction, in: Extended Abstracts of the 2004 Conference on Human Factors and Computing Systems, Vol. CHI 04. p. 1477. Available at: http://portal.acm.org/citation.cfm?doid=985921.986094.

[30] Palinko O Kun AL Shyrokov A Heeman P. (2010). Estimating cognitive load using remote eye tracking in a driving simulator, in: Proceedings of the 2010 Symposium on Eye-tracking Research & Applications. ACM, . p. 141licat.

[31] Erstenyuk V Swanson MR Siller M. Pupillary responses during a joint attention task are associated with nonverbal cognitive abilities and sub-clinical symptoms of autism. Res Autism Spectr Disord. (2014) 8:644–53. doi: 10.1016/j.rasd.2014.03.003 PMC4376279 25821516

[32] Reisinger DL Shaffer RC Horn PS Hong MP Erickson CA. Atypical social attention and emotional face processing in autism spectrum disorder: insights from face scanning and pupillometry. Front Integr Neurosci. (2020) 13. doi: 10.3389/fnint.2019.00076 PMC7026501 32116580

[33] Huang Q. A study of belief comprehension as a feature of psycho-theoretical development in children with autism. Master’s thesis, Hunan: Central South University (2014).

[34] Low J. Preschoolers’ implicit and explicit false-belief understanding: Relations with complex syntactical mastery. Child Dev. (2010) 81:597–615. doi: 10.1111/j.1467-8624.2009.01418.x 20438463

[35] Dang J King KM Inzlicht M. Why are self-report and behavioral measures weakly correlated? Trends Cogn Sci. (2020) 24:2674. doi: 10.1016/j.tics.2020.01.007 PMC7977810 32160564

